# Welcome to *Parasites & Vectors*

**DOI:** 10.1186/1756-3305-1-1

**Published:** 2008-01-07

**Authors:** Chris Arme

**Affiliations:** 1 Keele University, Keele, Staffordshire, ST5 5BG, UK

## Editorial

I am pleased to announce the launch of *Parasites & Vectors *– a new open access journal published by BioMed Central. The scope of *Parasites & Vectors *is wide, and encompasses all aspects of the biology of parasites, parasitic disease, intermediate hosts and vectors. Broader issues, such as economics, the social sciences and global climate change in relation to disease and disease control, will also be included. Two previous BioMed Central journals, *Filaria Journal *and *Kinetoplastid Biology and Disease*, are now incorporated into *Parasites & Vectors*, and I hope that the communities that supported these journals will also support this new venture.

*Parasites & Vectors *will be competing head-on with a number of existing subscription-based journals. However, I firmly believe that there is a niche for the new journal. The reason for this belief can be summarised in three words: 'online' and 'open access', both areas in which BioMed Central has great experience and an excellent track record.

*Parasites & Vectors *is published exclusively online. Articles will follow a consistent format so that the visual impact will be high and equal to that of the best hard-copy publications. In contrast to paper-based journals, however, the electronic format allows the full use of digital technologies and permits the inclusion of large data sets, from field and laboratory studies, links to other web pages, animations, slide shows, video clips and unlimited colour, all at no additional charge. Articles are published online on the day of acceptance and, soon after, listed in PubMed. To ensure permanency they are also archived in PubMed Central [1] and in digital repositories in Germany [2], France [3] and The Netherlands [4]. Open access means that all articles are freely available to all, worldwide, on the day of acceptance, and at no cost to the reader. Authors retain copyright of their work and can grant anyone the right to reproduce and disseminate it, provided that it is correctly cited and no errors are introduced.

In hard-copy journals, the costs of publication are met by subscriptions, paid by the reader. In *Parasites & Vectors*, as in other open access journals, these costs are borne by the author in the form of article processing charges (APCs). Many grant-awarding bodies recognise the value of open access publishing by allowing their funds to be used for APCs [5]. Institutes which have full membership of BioMed Central bear the cost of their author's publications [6], whilst a discount is available for institutions with supporting membership. Authors from countries that are in the World Bank's low income or lower-middle income categories may also have all their charges waived [7] and other individual APC waiver requests will be considered on a case-by-case basis. I shall make every effort to ensure that lack of funds does not impede my overall objective of publishing the best science, irrespective of authorship or country of origin.

I do not foresee that open access, online journals will totally replace the traditional print format in the immediate future, although this may be an increasing trend with time. I am certain, however, that the benefits of online publication, and the extra opportunities that digital technologies give to authors, will be increasingly recognised. Open access will transform the lives of scientists working in parts of the world where library facilities are restricted. BioMed Central is working closely with the *Institute for Scientific Information *to ensure that citation analysis of articles published in *Parasites & Vectors *will be available as soon as possible.

I believe that *Parasites & Vectors *will rapidly attract high quality manuscripts. Articles in *Parasites & Vectors *are peer-reviewed by at least two experts, drawn from the Editorial Board and the wider parasitological community. With the assistance of the Advisory and Editorial Boards [8] I shall ensure that the highest standards are maintained with a fast processing of manuscripts and fair refereeing and editorial decision-making. Of course, any new venture takes time to develop its own ethos and character. However, I am confident that the new journal will have soon attracted sufficient original research articles and reviews, to enable it to be judged a success.

For more information on *Parasites & Vectors*, its scope, refereeing policy and types of articles considered, please click on the link 'About *Parasites & Vectors' *at the top of this page. I look forward to receiving your manuscripts.
